# “ClusterApp”: A Shiny R application to guide cluster studies based on GPS data

**DOI:** 10.1002/ece3.11695

**Published:** 2024-07-22

**Authors:** Johanna Heeres, Aimee Tallian, Camilla Wikenros, Rick W. Heeres

**Affiliations:** ^1^ Department of Ecology Swedish University of Agricultural Sciences Riddarhyttan Sweden; ^2^ Norwegian Institute for Nature Research Trondheim Norway; ^3^ Department of Natural Sciences and Environmental Health University of South‐Eastern Norway Bø Norway

**Keywords:** animal activity, cluster analysis, fieldwork, movement data, Shiny application

## Abstract

The rapid evolution of GPS devices, and therefore, collection of GPS data can be used to investigate a wide variety of topics in wildlife research. The combination of remotely collected GPS data with on‐the‐ground field investigations is a powerful tool for exploring behavioral ecology. “GPS cluster studies” are aimed at pinpointing and investigating identified clusters in the field. Activity clusters can be based on various parameters (e.g., distance between GPS locations and the number of locations needed to establish a cluster), which are closely related to the set research questions. Variation in methods across years within the same study may result in data collection biases. Therefore, a streamlined method to parametrize, generate interactive maps, and extract activity cluster data using a predefined approach will limit biases, and make field work and data management straightforward for field technicians. We developed the “ClusterApp” Shiny application in the R software to facilitate a step‐by‐step guide to execute cluster analyses and data management of cluster studies on any species using GPS data. We illustrate the use of the “ClusterApp” with two location datasets constructed by data collected on brown bears (*Ursus arctos*) and gray wolves (*Canis lupus*).

## Graphical abstract

The evolution of GPS devices has revolutionized wildlife research by enabling the combination of GPS data with field investigations to explore behavioral ecology. GPS cluster studies focus on identifying and investigating activity clusters in the field based on specific parameters related to research questions. To address potential data collection biases from varying methods, the “ClusterApp” Shiny application was developed in R to streamline cluster analyses and data management, which is demonstrated with data on brown bears and gray wolves.

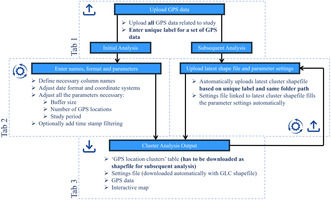

## 1 BACKGROUND

The advent of GPS‐tracking devices has revolutionized the world of wildlife tracking, helping researchers better understand the behavior of free‐ranging wild animals (Cagnacci et al., 2010; Kays et al., 2015). GPS data can be used to investigate a wide variety of topics ranging from movement ecology (Oleksy et al., 2019; Owen‐Smith et al., 2020) to animal space use or dispersal (Soanes et al., 2016; Tucker et al., 2018), foraging behavior (Kotzerka et al., 2010), and sociality (Albery et al., 2021). The combination of remotely collected GPS data with on‐the‐ground site visits is a powerful tool for exploring animal behavior (Cristescu et al., 2015). GPS data allow researchers to identify places where animals spend time on the landscape which can then be visited for further data collection. The collection of site visit data has refined our understanding of animal behaviors including diet and foraging patterns (Evans et al., 2016; Svoboda et al., 2013; van Dijk et al., 2008), fine‐scale habitat selection (Kusler et al., 2017; Schneider et al., 2013), reproduction (Moen et al., 2008), and context‐specific site choices (Bearman‐Brown et al., 2020; Siekiera et al., 2022).

GPS “cluster analysis” is a tool used to pinpoint areas of animal activity and select locations for in‐person site visits (e.g., Knopff et al., 2009; Ordiz et al., 2011; Sand et al., 2005). To define locations of interest, that is, GPS location clusters (hereafter clusters), researchers analyze movement patterns by defining and applying various parameters to GPS data to create clusters (e.g., distance between GPS locations, minimum number of locations, and time interval between GPS locations). After identifying the clusters, field technicians can visit the location to identify animal behaviors (e.g., denning, resting, foraging, or reproduction events), collect biological samples (e.g., scat and hair for diet and DNA analyses), record site characteristics (e.g., habitat type and cover), and collect a wide variety of other species or context‐specific data.

Many studies rely on a combination of geographical and statistical software such as ArcGIS (ESRI, 2023) and R (R Development Core Team, 2023) to generate clusters from GPS data. The algorithms used to generate clusters from GPS data can vary widely (see, e.g., Clapp et al., 2021), and the necessity for quick analysis in the field combined with field technicians' varied experience with geographical and statistical software could potentially lead to errors or biases in data collection. Additionally, a lack of standardization and detailed reporting of cluster parameters between different field seasons for the same data collection will lead to discrepancies in the data, which are later analyzed for scientific insights. Therefore, a standardized, easily reproducible method to generate clusters using a predefined approach may help limit errors and biases and make methodologies more transparent (Filazzola & Cahill, 2021). While well‐documented codes for clustering methods already exist (see “GPSeqClus” by Clapp et al., 2021 or “rASF” by Mahoney & Young, 2017), they require the users to have an understanding of R and coding. Therefore, we aimed to facilitate cluster analysis methodologies for the field technicians by using the Shiny R package (Chang et al., 2023). Shiny was developed to promote the assembly of easy‐to‐use applications using R software and provide a unique opportunity to create a user‐friendly application in this context.

Our goal is to provide an accessible, streamlined method to apply cluster analyses to GPS data for use in field‐based site visit studies. We aimed to make the in‐field cluster analysis as error proof as possible by keeping it outside of the programming environment. This approach clearly defines which inputs can be changed and what has to be kept untouched for the analysis to run in a standardized way, thus repeating the same analysis steps to develop activity clusters out of GPS locations within consecutive analyses on the same data. Importantly, the methods do not require users to have prior experience with geographical or statistical software. To facilitate this, we created the “ClusterApp” Shiny R application (version 1.0). The package that contains the application has to be installed into the R environment once, as with any other R package, and hereafter called the “library,” followed by the “run_app” command. We expect the application to contribute to the continuity of methods regarding data collection (e.g., decreased observer biases), offer straightforward usage for field technicians, and provide simple and reliable data management for research projects. Within a tutorial, we show the potential use of the application by applying it to datasets from two species, gray wolves (*Canis lupus*) and brown bears (*Ursus arctos*), and demonstrate how various research questions and species‐specific ecology affect the use of the application. Although our case study discusses large carnivore predation studies, the application can be broadly applied across a range of taxa and study objectives. This application was built using the “golem” framework (Fay et al., 2023), and is available as an installable R package. The application is continually under development and any updates will be communicated accordingly via the GitHub channel.

## 2 CONCEPT

The main goal of the “ClusterApp” is to simplify field logistics and standardize data collection and management for research projects utilizing GPS cluster analysis methodology for in‐field site visits. Note that it is important for users to establish their research questions and goals prior to use and to have a general understanding of the movement and behavior of their focal species to set functional parameters.

The application applies an algorithm to build clusters within a study period of interest by creating a buffer around each GPS location using a user‐defined distance. Buffers that spatially overlap within a user‐defined minimum number of locations will generate clusters. Variations in GPS device settings, such as fix rate (i.e., the time interval between GPS locations), or proximity/burst settings (i.e., increases in fix rate when the collar comes into proximity of another collar or defined area) can be handled by the application. The analysis can be run for one or multiple individuals and offers several ways to visually inspect the output data in the form of data tables and an interactive map displaying the results. This map allows interaction with the visualization as users are accustomed to GIS systems.

GPS cluster studies may require field technicians to visit GPS locations of focal individuals in almost real time which means they must find a balance between not disturbing the animal and visiting the site before evidence of the individual's activity disappears. Thus, researchers routinely download GPS data and run cluster analyses subsequently at regular, commonly short (e.g., every day or few days) intervals. It is, thus, standard practice that cluster analyses are run multiple times within one study period. In the context of data management, it is preferable to keep previously generated cluster IDs constant, that is, when new GPS data are added, all previous cluster IDs stay the same, while new clusters receive new, unique ID numbers. The application automatically loads previous shapefiles as well as the settings of the previous cluster analysis, meaning that cluster IDs stay unique and settings stay constant through the entire field season, allowing for streamlined data management and alignment. Additionally, the application offers the opportunity to manually adjust columns in the table or downloading a separate Excel sheet, allowing users to directly enter data collected in the field.

## 3 WORKFLOW

**FIGURE 1:**
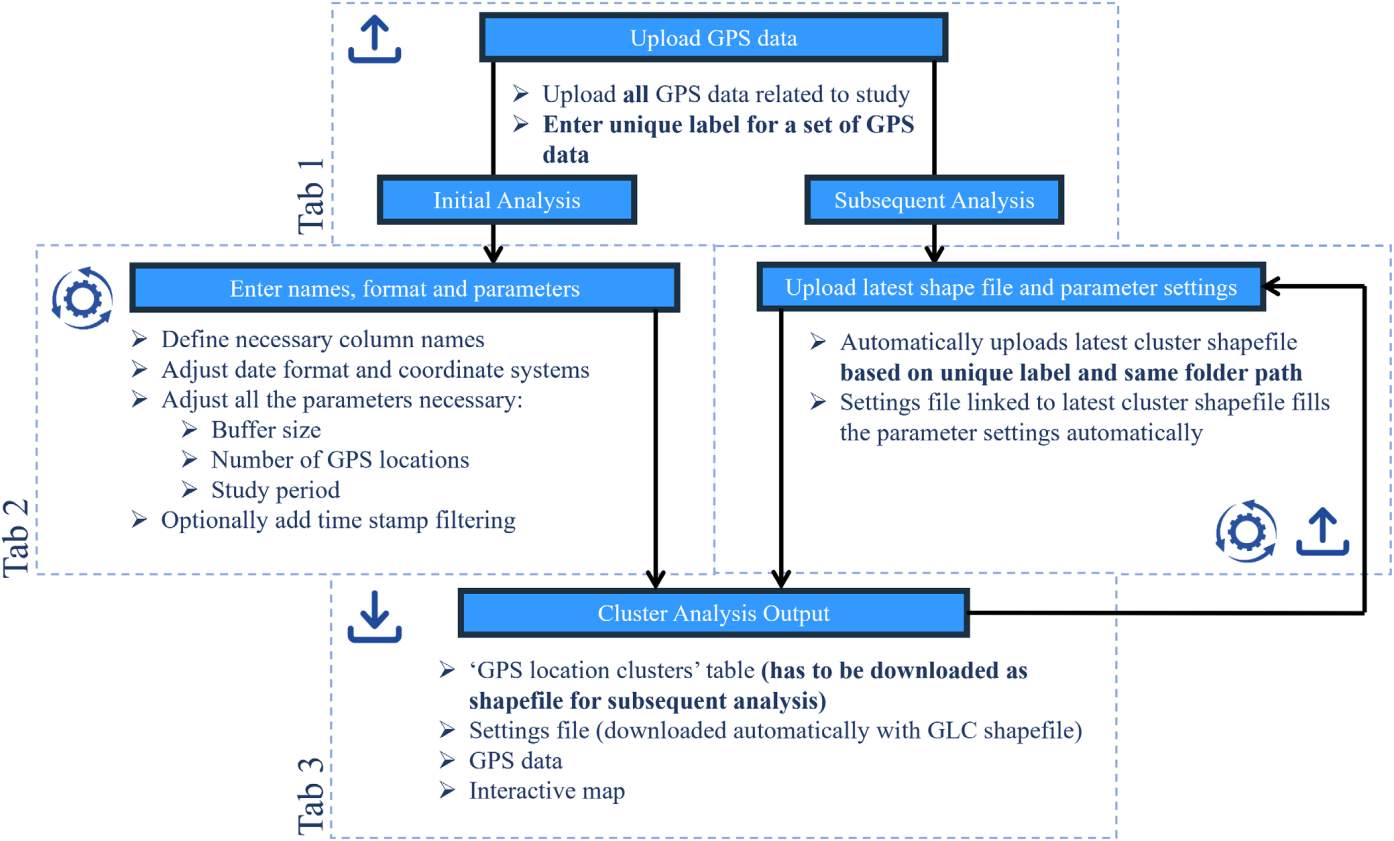
Flowchart showing the three main tabs when using the “ClusterApp” application to create clusters for investigations in the field.

The application has three main steps (Figure 1) available as “tabs” in the left panel of the application. Each tab has several sub‐steps, and the tabs can be freely navigated between. The first tab allows the user to upload data files, define data formatting, and add a unique label to the cluster study. The second tab allows users to define the cluster methodology, which affects the cluster analysis output. Within the initial analysis (i.e., the first analysis that is performed with a given dataset), the parameters have to be entered, while in all other subsequent analyses (i.e., any additional analysis that is performed with additional GPS data to the given dataset), the cluster analysis output, specifically the settings and latest cluster files, fills the parameters automatically. The third tab allows the user to run the cluster analysis and display and download the results in the form of tables and an interactive map. Within the application are built‐in guidance options marked by blue information icons, which can be accessed by hovering over them.

### 3.1 Running the analysis

#### 3.1.1 Tab 1. Upload GPS data

The raw GPS file used for data upload can be in the following file formats: .*shp* or .*csv*. The windows “Data” and “Data Summary” provide information about the uploaded data which can be used to check if the data were uploaded correctly and identify relevant column names. Additionally, a unique label for the set of GPS data must be defined; this label must stay the same throughout a study period.

#### 3.1.2 Tab 2. Adjust cluster analysis parameters

**FIGURE 2:**
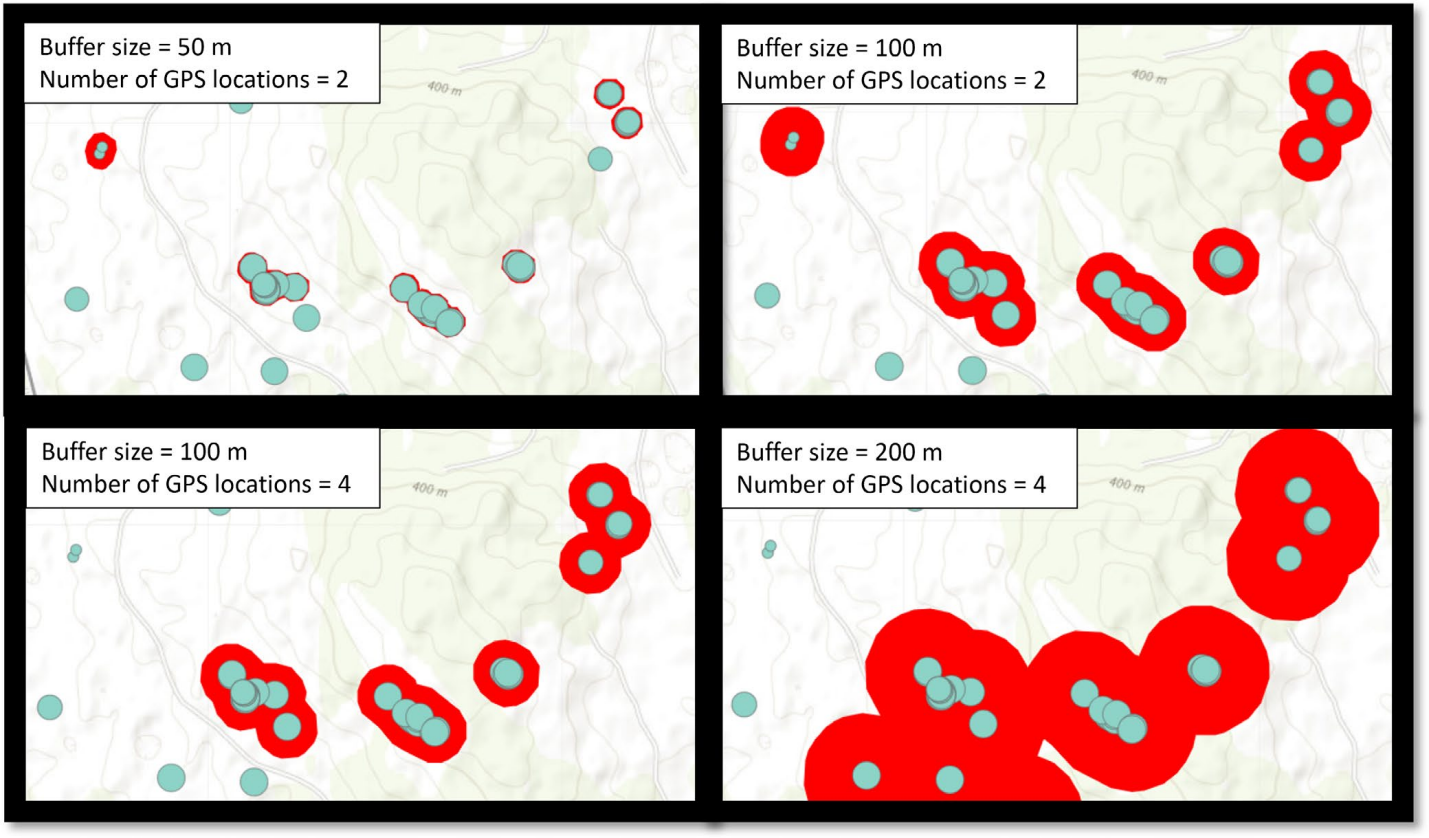
Changes to the size of the buffer (m) or the minimum number of GPS locations influence the cluster output (red polygons) in the “ClusterApp” application. Turquoise points show the same selection of GPS locations of one collared wolf in each of the four panels.

One of the important tasks with cluster analyses, which is closely related to the predefined research questions, is to select the parameters necessary for identifying biologically relevant clusters. These parameters must stay the same throughout the study period in order to generate comparable clusters. They are formed by applying a buffer of *x* meters around every GPS location. If (a) these buffers overlap (so at a maximum distance of *x* × 2), (b) there are *y* numbers of GPS locations within these overlapping buffers, and (c) the buffered GPS locations are within the study period, then they will generate a cluster. The specific research question and therefore the criteria for how a GPS cluster is developed have to be defined beforehand, as the chosen parameters can affect number and size of the resulting clusters as well as the subsequent inference made on the data collected (Cluff & Mech, 2023; Figure 2).

Additionally, the option “Should only clusters with consecutive GPS locations be used?” will generate clusters for only *y* number of GPS locations that are sequential in time and within the defined buffer distance of *x*. Buffers around GPS locations can therefore overlap with another, yet not develop a cluster if they are not sequential in time. Furthermore, optional adjustments include filtering the GPS data to only include locations every *z* minutes to account for irregular fix intervals by, for example, proximity or burst events. The default is to use all available GPS data, which can introduce bias if there are frequent changes in the fix rate of the GPS devices. The user can, further, decide whether columns with information on time spent at the location and the number of locations within/outside of the cluster should be added to the final table in the third tab. This only serves for overview purposes.

The third column in this tab relates to the subsequent analyses that are done after the initial analysis. A common procedure during fieldwork includes visiting clusters that were made by the most recent GPS locations in real time. In order to keep earlier generated cluster IDs constant, even with additional locations possibly increasing, combining, or creating new clusters, and to keep the data management up to date, the output of an earlier cluster analysis has to be downloaded as a shapefile.

When entering the same label as for the initial analysis in tab 1, the app will automatically search and upload the cluster shapefile within the file path of the loaded GPS data. If the analysis is run for the first time or the app does not detect any shapefiles, a message will read “No latest cluster file” and will run the analysis as if done for the first time (= initial analysis). If a previous shape file is found, the path will appear, and the associated settings will be filled in automatically (= subsequent analysis). Optionally, all clusters from the old cluster file can automatically be marked as “Done” upon import.

#### 3.1.3 Tab 3. Cluster analysis output

The cluster analysis is performed by clicking the button “Perform Cluster Analysis” under the third tab “Cluster Analysis Output.” If the analysis runs successfully, the output will appear as two data tables in the windows “GPS location clusters” and “GPS data.” Furthermore, the data can be displayed on an interactive map by clicking on “Plot data.”

### 3.2 Working with the analysis output

#### 3.2.1 “GPS location clusters” table

The “GPS location clusters” table appears first as the default setting and contains all the data concerning the clusters generated by the analysis. Table columns include the “Animal ID” (selected as “Animal ID” column during data upload), the unique “Cluster ID” (built as “Animal ID” underscore “Cluster ID”), and relevant information regarding each cluster (e.g., percent of time spent at the cluster and number of GPS locations inside and outside the cluster during the first and last date of visit at the cluster, and mean center locations). Data can be manually adjusted for the following columns: “State” (if the cluster has been visited or grown since the last cluster analysis), “Event” (what was found at the location), “Date Done” (when the cluster was visited), “Field technician” (who visited the cluster), and additional “Notes.” Adjustments for the columns “State” and “Event” can change the aesthetics of the interactive map (see Section 3.2.3), facilitating streamlined field logistics and data management. Searching and filtering options allow for a user‐friendly way to search and export specific output. Downloading the cluster file as a shapefile is mandatory for changes in data entry to be saved and for the cluster output to be used in subsequent analyses.

#### 3.2.2 “GPS data” table

The “GPS data” table gives an overview of all GPS locations that were used for the cluster analysis. Columns include “Animal ID,” “Point ID,” “ClusterID,” timestamps, and the spatial coordinates. “Point ID” is a unique identifier for each GPS location, represented by a combination of “Animal ID,” the cluster number it belongs to or single point (SP), and the month, day, and hour of the GPS location.

#### 3.2.3 Interactive map

**FIGURE 3:**
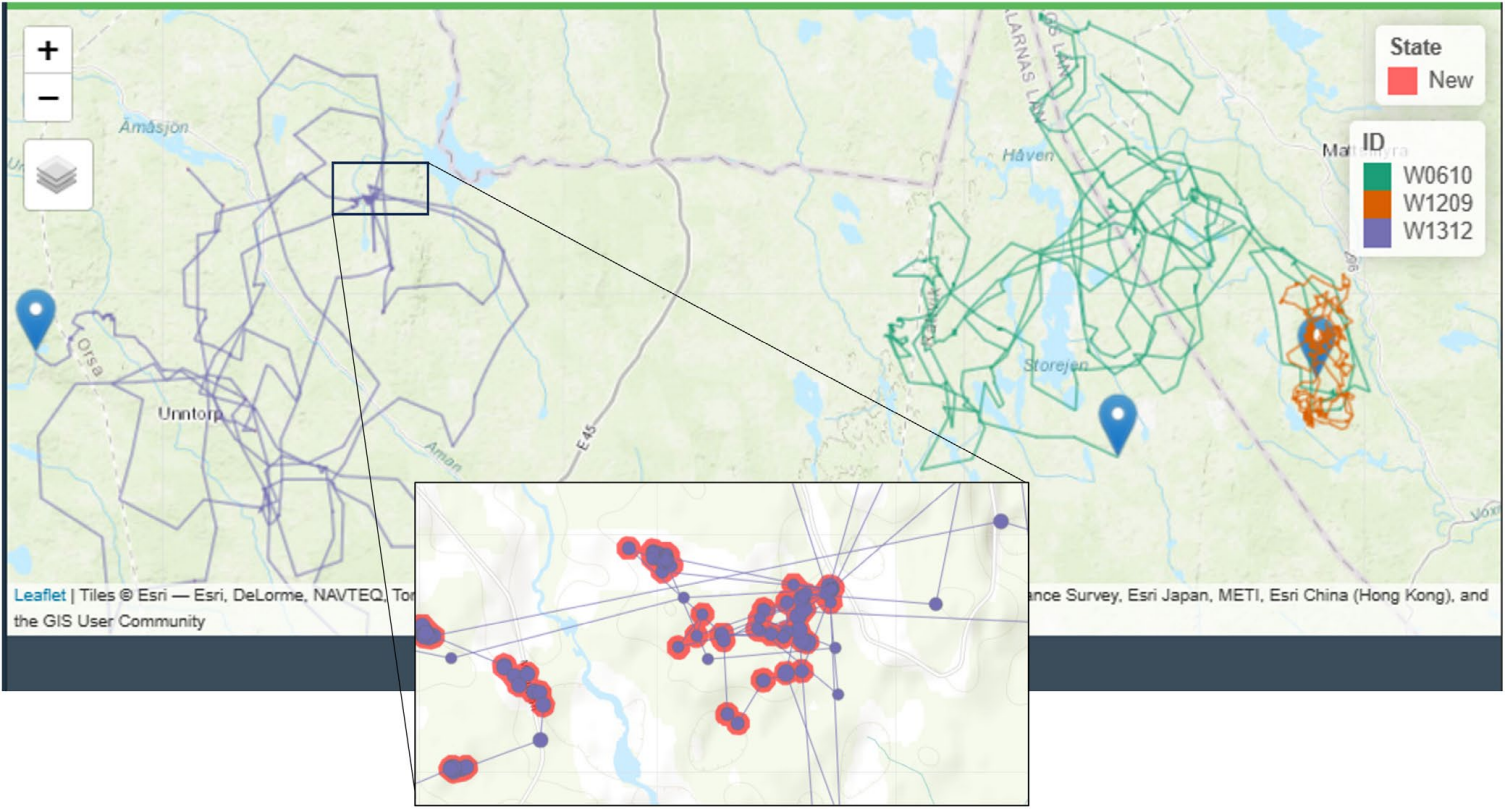
Examples of the visualized clusters within the interactive map in the “ClusterApp” application. Cluster polygons are filled according to their “State” (in this example, all red for State = New), while the track and GPS locations of the three brown bears used in this example are visualized with different colors. The blue pin drops show the last position for each brown bear.

The “ClusterApp” has the option to create an interactive map that allows users to visually inspect relevant cluster data (Figure 3). The interactive map will appear below the “GPS location clusters” table by clicking the “Plot Data” button. The map displays several layers from the cluster analysis output; the default display shows cluster polygons colored according to the “State” column, as well as the most recent GPS locations of the collared individual(s). Additional options that can be displayed include GPS locations, GPS tracks, cluster ID labels, and the “Events” for clusters. GPS locations increase in size as they become more recent in time, while GPS tracks display a line between consecutive locations in time for each individual. “Events” shows cluster polygons filled according to the data entered in the “Event” column. These options help visualize the study area, for example, locating clusters that have yet to be visited and identifying what was found in clusters that were already evaluated. Furthermore, when using the filtering option with the GPS cluster table, only this selection of clusters will be shown when plotting the data anew. By selecting one row of the cluster table and plotting the data anew, this cluster is highlighted in the map with a light‐yellow pointer. These and many more options explained within the tutorial (see Section 5) allow for user‐friendly exploration of the spatial distribution of specified clusters.

#### 3.2.4 Downloading output

All outputs from the “GPS location clusters” and “GPS data” tables can be downloaded in several formats: shapefiles (.*shp*) offer the opportunity to use the spatial data in other contexts (e.g., GIS software), Excel files (.*xlsx*) make data management outside of the application possible, and GPS exchange format files (.*gpx*) can be loaded directly on hand‐held GPS devices. The shapefile will download all data, while the .*xlsx* and .*gpx* files will only download the filtered table if filters were used. Furthermore, the interactive map can be exported as an interactive web link (.*html*).

## 4 CASE STUDY

**FIGURE 4:**
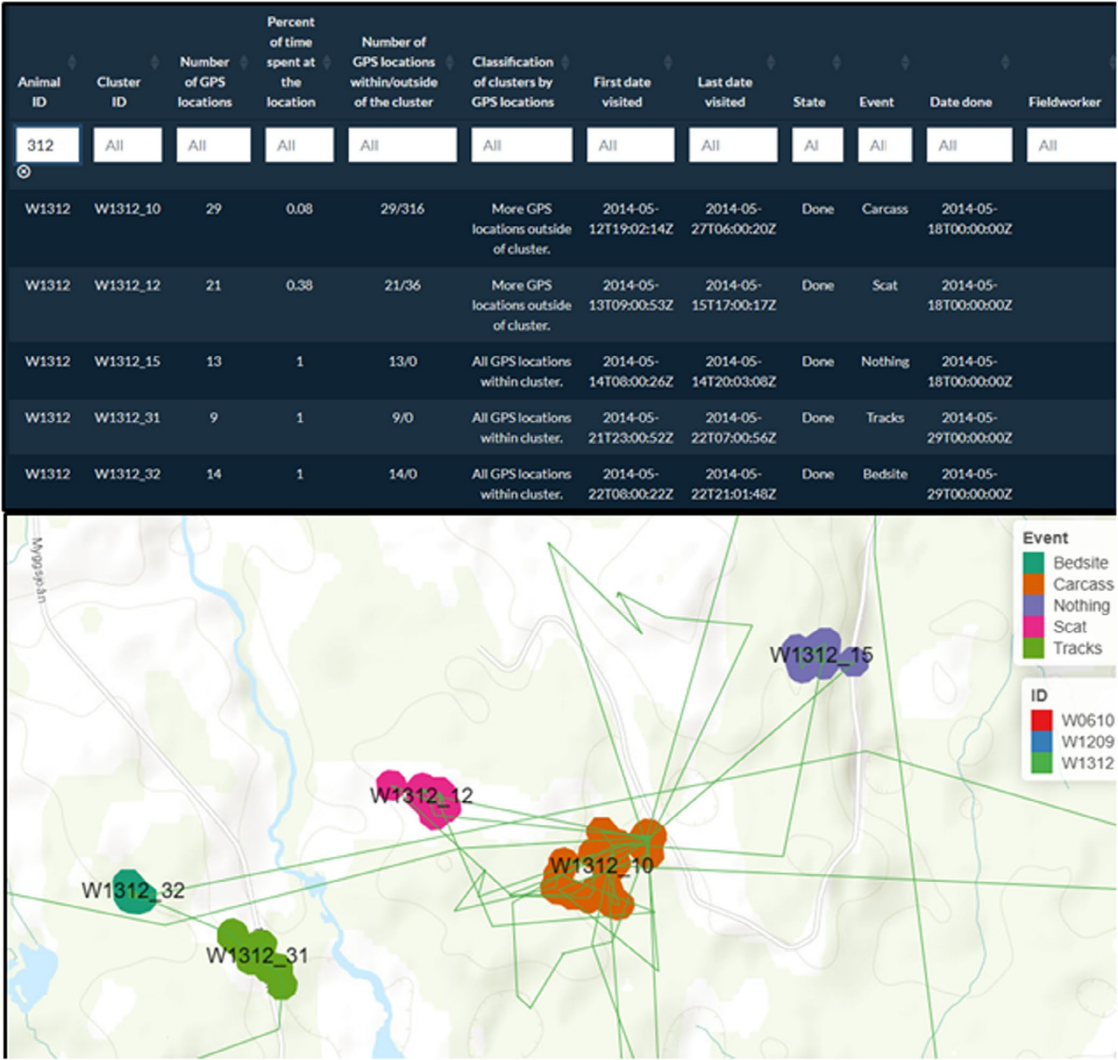
Completed data table (filtered for the individual W1312) and visual output of the clusters in the “ClusterApp” application, which are filled according to the “Event” column. After on‐the‐ground site visits of the identified clusters, this column, as well as the columns “State” and “Date done” could be filled in with an array of identified activities at the clusters.

We show the use of the application and the results of a real on‐site cluster study conducted on several individuals using data for brown bears followed by the Scandinavian Brown Bear Research Project (SBBRP). Firstly, the brown bear data include the GPS locations of three individuals that were followed from May 1 to May 31, 2014 (Ordiz et al., 2020). The goal of this study was to assess brown bear–moose (*Alces alces*) predation while collecting data on other relevant brown bear behavior. Clusters were generated using 30 m buffers with a minimum of two GPS locations (Ordiz et al., 2020; Rauset et al., 2012). By applying the clustering method and visiting them on‐site, field technicians detected an array of different activities and signs such as carcasses, bed sites, tracks, and scats (Figure 4).

For these three individuals, the GPS fix intervals were constant at ~60 min, so all GPS locations were used for the analysis. As all GPS locations were collected at the same time interval, the clusters generated for each individual are comparable with each other. However, some research projects may have GPS data that were sent at different time intervals, for example; changing fix rate schedules between day and night, seasonal changes, or general data set up (Wikenros et al., 2023). Additionally, collars with proximity sensors are increasingly being used (Drewe et al., 2012; Kirkpatrick et al., 2021; Le Grand et al., 2019; Ripperger et al., 2020). These can be used to, for example, detect interactions between individuals of the same or between different species, as, for example, done by Tallian et al. (2023). They used proximity sensors to detect interactions between collared brown bears and moose individuals in Sweden. The brown bear data, therefore, secondly, include GPS locations of the single brown bear followed by this study. The cluster that developed during a GPS burst identifies a predation event of a moose calf, whose mother stays in the proximity of the event (for detailed results, see Tallian et al., 2023).

## 5 TUTORIAL

To facilitate streamlined and accurate implementation for first‐time users and highlight the main features of the “ClusterApp,” we encourage the use of the “Tutorial” (available and being updated with new versions on GitHub as well as a “vignette,” which is part of the installable R package). The tutorial uses data from two large carnivore species in Scandinavia (available as part of the R package) and includes guidance on all cluster analysis steps as well as specific data‐based adjustments (e.g., proximity events). The chosen cluster parameter settings within the tutorial are based on real cluster studies conducted on the individual(s) within the GPS data sets provided by the Scandinavian Wolf Research Project (SKANDULV) and SBBRP (Ordiz et al., 2020; Tallian et al., 2023; Wikenros et al., 2023). To demonstrate the basic use of the application and how to execute initial and subsequent cluster analyses, we used data from a GPS‐collared wolf from the SKANDULV project. To clarify the use of the application for applying cluster analysis to multiple individuals in the same input file, we used data from three GPS‐collared brown bears followed by the SBBRP. We also show how to recognize and handle GPS burst data from proximity events using data from an individual brown bear followed by the SBBRP.

## 6 FINAL REMARKS

GPS cluster‐based research projects navigate an array of logistics surrounding complex data handling and management techniques when conducting fieldwork. Automating the repetitive task of running cluster analysis multiple times per study period due to the constant flow of new data saves time and effort. Streamlining and conducting GPS cluster analysis systematically in the programming language R decreases the chance of error during field data collection. It also allows projects to maintain methods across field technicians and seasons and makes field methodology simple, transparent, and reproducible, which are all important within the process of science and a basic requirement for the advancement of ecological research (Cassey & Blackburn, 2006; Filazzola & Cahill, 2021). The “ClusterApp” combines code‐based, therefore reproducible, workflows as implemented in R, while the user‐friendly interface of the Shiny applications allows for the simple implementation for field technicians.

While the R environment offers an undoubted ability to flexibly analyze data and tailor cluster code to the researchers' specific needs, it is our experience that field technicians who are not always practiced with geographical or statistical software shy away from doing their own cluster analyses. Additionally, by only making a function available within the R environment, mistakes are prone to happen when, within the process, it is not clearly defined which inputs are supposed to be changed and which parameters should be left untouched. Yet, it is essential that cluster study fieldwork is an integrative process, where the field technician doing the fieldwork is also in charge of doing the cluster analyses. Thus, the “ClusterApp” was developed primarily for the field technicians using the user‐friendly interface that Shiny applications provide.

The potential limitations of the application arise from the aforementioned strengths. For example, because the code is packed in a user‐friendly interface, the code running in the background cannot be accessed or adjusted. This makes troubleshooting errors more complicated if the users are not experienced with Shiny coding. However, the application is built around one main function producing the cluster analysis output (which is available in “Chapter 6: Main Function” within the tutorial). For more experienced R users, troubleshooting of any data‐related issues leading to errors in the implementation of the cluster analysis function can, therefore, be done within the R environment as needed. Another limitation might arise from encouraging field technicians to independently generate clusters, which may create traceability issues if errors in the data are found later on. However, when downloading a cluster shapefile, the application also automatically downloads a settings text file stating all the chosen input parameters. These settings files can be saved and used later to retrace and understand any possible errors or mistakes in the data.

Well‐documented codes for generating clusters from GPS data, such as the “GPSeqClus” package, exist within R. The “GPSeqClus” package “provides an efficient data processing routine to build, characterize, visualize, and navigate clusters” from GPS data (Clapp et al., 2021). It follows a clustering method by first searching for nearby GPS locations within a defined time and distance and assigning them to the same cluster. For each cluster of GPS locations, it identifies a “stay point,” which is the centroid of the combined GPS locations. This “stay point” is recalculated as new GPS locations are added to the clusters. The clustering algorithm developed within our approach differs slightly from the method used in the “GPSeqClus” package but follows common methodology used in a variety of other studies (Ordiz et al., 2020; Wikenros et al., 2023). We see future potential in integrating different clustering algorithms, such as the functions of the “GPSeqClus” package into the application to allow users to choose the clustering method that best fits their research project and question.

Overall, we believe that the “ClusterApp” fills a gap in the iterative GPS data clustering procedures commonly employed to direct on‐the‐ground site visits and data collection used to explore animal behavior. Data analysis is simple, quick, and reproducible and the integrated interactive GIS tools allow for easy exploration of the clusters. This simultaneously saves time for field technicians while standardizing the clustering methods used by a project within and across seasons. Clusters can be easily labeled as completed or not, and events regarding what was found can be entered and visualized; this streamlines workflows in the field, puts observations at clusters in biological context, and helps minimize errors due to missed cluster visits. Furthermore, analysis settings can be examined within the output files, which allows field technicians and researchers the ability to retrace methodologies. The standardized repeatability and reproducibility of cluster analysis methods during fieldwork using the “ClusterApp” are fundamental for the process of science and will help advance our understanding of the behavior of free‐ranging wild animals.

## AUTHOR CONTRIBUTIONS

**Johanna Heeres:** Conceptualization (equal); formal analysis (equal); methodology (equal); writing – original draft (equal). **Camilla Wikenros:** Data curation (equal); funding acquisition (equal); writing – review and editing (equal). **Aimee Tallian:** Data curation (equal); funding acquisition (equal); writing – review and editing (equal). **Rick W. Heeres:** Conceptualization (equal); project administration (equal); writing – original draft (equal).

## FUNDING INFORMATION

The Scandinavian Wolf Research Project was funded by the Swedish Environmental Protection Agency (“Viltvårdsfonden” grant nos. 2021‐00025 and 2022‐00102, and “Basverksamhet för SKANDULV” grant no. 328‐22‐003). The Scandinavian Brown Bear Research Project was funded by the Swedish Environmental Protection Agency ("Viltvårdsfonden” grant no. 2022‐00102) and the Norwegian Environmental Protection Agency.

## CONFLICT OF INTEREST STATEMENT

The authors declare no conflict of interest.

## Data Availability

The datasets used in the manuscript are accessible within the R package. The source code and installation manual of the ‘ClusterApp’ application is available on https://github.com/JohannaMz/ClusterApp.
